# Phrasal Learning Is a Horse Apiece: No Recognition Memory Advantages for Idioms in L1 and L2 Adult Learners

**DOI:** 10.3389/fpsyg.2021.591364

**Published:** 2021-04-15

**Authors:** Sara D. Beck, Andrea Weber

**Affiliations:** ^1^SFB 833, University of Tübingen, Tübingen, Germany; ^2^Psycholinguistics and Applied Language Studies and SFB 833, English Department, University of Tübingen, Tübingen, Germany

**Keywords:** idiom, memory, recognition, non-native (L2) speaker, phrasal learning, literal and figurative

## Abstract

Native (L1) and to some extent non-native (L2) speakers have shown processing advantages for idioms compared to novel literal phrases, and there is limited evidence that this advantage also extends to memory in L1 children. This study investigated whether these advantages generalize to recognition memory in adults. It employed a learning paradigm to test whether there is a recognition memory advantage for idioms compared to literal phrases in adult L1 and L2 learners considering both form and meaning recognition. Additionally, we asked whether the presence of unfamiliar vocabulary interferes with phrasal learning by looking at recall of such unfamiliar words. When encountering new idioms, L2 learners often must cope with both figurative meaning and unfamiliar vocabulary. While single word meaning need not interfere with idiomatic meaning, it is a building block for the meaning of literal phrases. In Experiment 1, L2 learners showed equal recall for the form and meaning of literal and idiomatic phrases in which either all words were highly familiar, or one word was unfamiliar. However, unfamiliar words decreased overall recognition and were also remembered significantly better in literal compared to idiomatic phrases. In Experiment 2, L1 speakers also showed no recall differences between phrase types, but they displayed a trending increase in recognition in the presence of unfamiliar words. We conclude that there is no inherent recognition memory advantage for idioms based on figurativeness alone, and word- and phrasal meaning interact differently in learner groups.

## Introduction

Idioms challenge language learners as they represent a figurative meaning that is not achieved through classic language composition. *To shoot the breeze*, for example, means to have an informal conversation, and neither the meaning of “shoot” nor “breeze” contributes straightforwardly to this meaning as is the case in compositional literal language. Rather, in order to understand the idiom, one must often simply learn the definition, and this holistic meaning may be associated with the idiomatic phrase or unit in a word-like manner (e.g., Wray, 2002). While there are also idioms that are arguably more transparent in meaning, in that individual words make contributions (e.g., *hit the mark*), non-transparent idioms, such as *shoot the breeze*, will be the focus of this study. In both cases, not only are adult native (L1) and highly proficient non-native (L2) users of a language able to interpret idioms quickly and without difficulty (e.g., Beck and Weber, 2016a), but they also may be able to do so more quickly than with comparable novel phrases (e.g., Underwood et al., 2004). Additionally, these processing advantages seem to extend to recognition memory in native children (Reuterskiöld and Van Lancker Sidtis, 2012). That is, newly learned idioms may be remembered better than comparable literal phrases. However, the extension of this processing advantage to recognition memory has not yet been examined in adults, neither for L1 language users nor L2 language learners. In addition to storing and accessing the phrasal meaning signaled by the idiomatic unit, individual words can present additional challenges for language learners, particularly when they are unfamiliar with them (e.g., *breeze* in *shoot the breeze*). If single word meanings play a role in idiom recognition, then unfamiliar words may cause phrasal learning to be affected by the presence of such words, and, in turn, single word-learning to be negatively affected in idiomatic phrases.

In the current study, we compared the learning of figurative and literal phrases. In contrast to previous studies using newly learned phrases, many of which compared different phrases, identical phrases were used and either learned with an idiomatic or a literal meaning. This allowed us to preclude an influence of structural or lexical differences between phrases on the outcome. The study addressed three questions in two experiments: 1. Is there a recognition memory advantage for newly learned idioms compared to literal phrases for L2 learners? 2. Does learning unfamiliar words and phrases interact and affect recognition memory? 3. Do adult native speakers show a comparable recognition memory advantage for newly learned idioms and identical literal phrases? Because of the novelty of these questions, the literature discussed below will address studies that motivate the current study including idiomatic processing, memory, and learning studies as well as studies addressing the interaction between word- and phrasal memory.

### Idiomatic Processing and Memory

Though there are few studies dealing directly with memory for idioms compared to similar novel phrases, there is a multitude of literature dealing with idiomatic processing and how it may compare to novel, literal language processing. Idiomatic processing theories vary widely but generally take basis in the idea that idiomatic meaning is at least partly non-compositional. Although theories of processing attempt to explain the presence of the so-called *idiom superiority effect*, which describes the fast nature of idiomatic processing compared to other types of phrases, there is not a consensus on how this process occurs. However, recent psycholinguistic literature favors hybrid approaches to idiomatic processing (e.g., Cacciari and Tabossi, 1988; Sprenger et al., 2006) which consider the interaction and possible competition between literal and figurative meaning over step-wise approaches (e.g., Bobrow and Bell, 1973; Swinney and Cutler, 1979) which prioritize ordered procedures in meaning access and storage. Such hybrid approaches are supported by consistent evidence that processing occurs quickly, and without cost in advanced language users while also remaining flexible and subject to linguistic influence, for example from idiomatic properties and context (e.g., Titone and Connine, 1994; Titone and Libben, 2014; Beck and Weber, 2016a, 2020).

When compared to similar novel (literal) phrases, idioms consistently show processing advantages in reading (e.g., Gibbs, 1980; Conklin and Schmitt, 2008; Siyanova-Chanturia et al., 2011). Underwood et al. (2004) looked at eye-movements during the reading of passages containing embedded idioms compared to novel phrases. Native speakers fixated less on the final constituent word of an idiom compared to the same word in a non-formulaic context (e.g., “teeth” in *met the deadline by the skin of his teeth* vs. *the dentist looked at his teeth*), and the duration of fixations was also shorter. In a self-paced reading paradigm, Conklin and Schmitt (2008) also found that reading times were shorter for idiomatic phrases compared to more closely matched controls (e.g., *hit the nail on the head* vs. *hit his head on the nail*). Siyanova-Chanturia et al. (2011) replicated these results with eye-movements and found that not only were fixations shorter on idiomatic phrases, but first-pass and total reading times were also shorter than matched control phrases. Interestingly, however, the latter two studies also compared literal and figurative readings of these familiar idioms and found that native speakers did not show any differences between the two readings. Thus, it appears that the processing advantages associated with speed of reading are not associated solely with figurative uses alone. Rather, these advantages may stem from familiarity with the phrase. However, both the literal and figurative readings compared in this case were associated with familiar phrases and not for newly learned or novel phrases, so here, too, it is unclear how novel idioms and equivalent literal phrases stack up. Decidedly, however, if figurativeness rather than familiarity does play a role in the advantages associated with idiomatic processing, then this advantage may also extend to other processes such as memory.

This processing advantage does appear to extend to recognition memory for idioms in native speakers. Reuterskiöld and Van Lancker Sidtis (2012) looked at young girls' retention of idioms after a single exposure in conversational speech compared to novel literal phrases. They found that both age groups tested (8–9 and 12–14 years) scored higher on recognition and comprehension of target idioms compared to literal phrases and non-target idioms. Authors concluded that idiomatic phrases, unlike literal phrases, are acquired holistically, allowing for a more rapid retention compared to similar literal phrases. These results are generally supported by earlier memory studies conducted on idiomatic or unitized word pairs suggesting that idiomatic word pairs are represented in a holistic, unitized manner (e.g., Horowitz and Manelis, 1973; Schachter and McGlynn, 1989). In one such study, Schachter and McGlynn (1989) compared implicit memory for unitized (idiomatic) word pairs (e.g., sour grapes) to non-unitized word pairs (e.g., soft soap). In free association tasks, implicit recall of non-unitized word pairs was improved by all elaborative study measures (e.g., defining tasks and synonym naming) whereas such improvement was not seen in all elaborative conditions for the unitized pairs. The differences between these types of word pairs support the view that idioms are represented holistically, unlike the other word pairs, and processing and memory advantages may be a result of such storage. Unclear, however, is both whether these processes are as immediate, as suggested by the Reuterskiöld and Van Lancker Sidtis (2012) study, and whether this effect is related to the figurative meaning associated with idioms or rather their formulaic nature. The paucity of further studies on memory for idioms suggests that additional investigation is needed.

Unlike in native speakers, there is mixed evidence that processing idioms is faster than novel literal phrases in non-native speakers, and no research on memory for idioms to our knowledge. L2 readers were examined in Underwood et al. (2004), Conklin and Schmitt (2008), and Siyanova-Chanturia et al. (2011), and each found slightly different results. Underwood et al. (2004) found that while L2 readers fixated less on final constituent words in idioms compared to non-formulaic phrases, the duration of these word-final fixations was not shorter as was the case in L1 readers. Considering reading times of such phrases, however, Conklin and Schmitt (2008) found consistent advantages for idioms compared to controlled novel phrases in both figurative and literal contexts, just as in L1 readers. However, Siyanova-Chanturia et al. (2011) were unable to replicate these results using eye-tracking. Not only did L2 readers show no differences in the number of fixations, first-pass, and total reading times between idioms and controlled novel phrases, unlike their L1 counterparts, but figurative readings of idioms were read more slowly than literal readings. Following these results, figurativeness may even place additional processing burdens on L2 readers. Critically, however, each experiment used different methods to test previous knowledge of the idioms, and the extent to which the given participants in the studies were familiar with the idioms used was not directly verified nor can variability in familiarity be entirely ruled out. In any case, idiomatic units may not carry the same processing advantages for L2 speakers present for L1 speakers, and their figurative nature seems to present additional processing challenges.

While we are unaware of any existing L2 recognition memory studies dealing with idioms compared to novel literal phrases, there is a plethora of literature focusing on how to increase learning of and memory for idioms in L2 learners (e.g., Iulian, 2018; Miller, 2020). Although the general challenges of learning idioms are well-documented (e.g., Cooper, 1999), there are a number of techniques that increase L2 idiom learning. Processes that improve learner recall of idioms and other multi-word units include, but are not limited to, noticing exercises (e.g., Boers et al., 2006), typological enhancement (e.g., Peters, 2012), the use of dictionaries and look-up tasks (e.g., Laufer, 2011), translation exercises (e.g., Laufer and Girsai, 2008), and focus on sound repetition (e.g., Boers and Lindstromberg, 2005). Additionally, idiom-specific differences such as imageability and transparency may also play an important role in their retention (e.g., Steinel et al., 2007; Cucchiarini et al., 2020). For example, Steinel et al. (2007) investigated receptive and productive learning of English idioms in Dutch learners and found that highly imageable idioms, or those easily pictured mentally (e.g., *keep a straight face*), have an advantage in receptive learning over those less easily pictured (e.g., *hang fire*). Additionally, they found that transparency, the overlap between figurative and literal meaning, also affected recognition. Tiv et al. (2016) also investigated the effect of transparency as well as ambiguity (whether both literal and figurative interpretations are possible) in a training study using L1 English speaking adults learning translated French idioms. Using elaborative training procedures and a cued recall task in two experiments, results suggested that highly transparent idioms were recalled better than less transparent idioms. Ambiguity and individual differences (i.e., O-Span task), however, did not affect learning outcomes. Finally, in a recent study using learners of Dutch idioms, Cucchiarini et al. (2020) confirmed that transparent idioms outperformed non-transparent idioms in an L2 CALL environment with intense practice. Interestingly, these studies also showed that L2 learning is successful even with only few exposures to new idioms. These studies, however, investigate idiom-learning separately from other types of phrasal learning (i.e., literal phrases). Thus, while comparisons between idioms and similar control phrases in L2 memory are lacking, elaborative processes are helpful, and some idioms may have inherent memory advantages over others.

### Phrasal- and Word- Competition

Idioms are unique from other types of formulaic phrases in that their phrasal (figurative) meaning is not necessarily derived from the meaning of the constituent words, and this meaning may be stored holistically (e.g., Wray, 2002). While holistic representation could explain the processing advantages described above, it could also present a disconnect between activation of the meaning of the individual constituent words and the phrasal meaning. Where individual word meaning is unnecessary, there is evidence that native speakers may interact less with literal constituents. For instance, processing studies in L1 speakers show that literal constituent meaning need not be activated where context or highly predictable phrases are presented (e.g., Titone and Connine, 1994; Rommers et al., 2013). Additionally, looking at memory for unitized word pairs, Horowitz and Manelis (1973) found evidence that recognition of individual words in idiomatic pairs was worse than recognition in non-unitized word pairs (e.g., *cold war* vs. *cold egg*). However, these constituents seem to remain relevant during processing. For example, a study investigating the effect of word frequency on the phonetic duration of words in highly frequent multi-word units suggests that even in formulaic units, such as idioms, the frequency of individual words continues to have effects on the entire phrase (Arnon and Cohen Priva, 2015). The authors suggest that such units need not be represented holistically since constituent words retain their ability to affect the phrase. Thus, where phrases with equal frequency are compared, whether literal or figurative, it may be the case that the processing effects following both word- and phrasal-meaning remains. This idea is also substantiated by evidence that both L1 and L2 speakers still activate both literal constituent and figurative meanings in various online processing circumstances (e.g., Beck and Weber, 2016a,b).

However, there is some debate as to whether L1 and L2 speakers diverge in the importance of constituent word meanings during idiom processing. Not only do learners tend to neglect the learning of phrases compared to individual words (e.g., Durrant and Schmitt, 2009), but L2 speakers may rely more on literal constituent meanings during language processing as a whole because of the saliency of literal word meaning in L2 language use and learning (see e.g., Cieślicka, 2006 and Giora, 1997). This claim receives indirect support from offline idiom studies in which there is evidence that learners use the constituents of idioms to build meaning, even where meaning does not directly contribute to the meaning of the phrase (e.g., Abel, 2003a,b). Learners also consistently rate idioms as more transparent than native speakers do (Hubers et al., 2020). Online evidence for this tendency in L2 idiom processing can be seen in cross-modal priming results from Cieślicka (2006) in which priming was greater for literal constituents of an idiom compared to the figurative meaning of the phrase. Later studies using L1 control participants, however, do not support this conclusion (Beck and Weber, 2016a; van Ginkel and Dijkstra, 2019). Thus, it is unclear whether the results from Horowitz and Manelis (1973), in which constituent words were recalled better in non-idiomatic compared to idiomatic word pairs would also apply to L2 speakers. Additionally, experimental methods looking at meaning activation in idiom processing have focused on highly familiar idioms and assumed vocabulary knowledge for all constituent words. For L2 speakers, however, it may be the case that an idiom is both unknown as a phrase and contains unfamiliar constituent vocabulary. This competition between word- and phrasal-meaning may have consequences both on the memory for the phrase as well as the memory for the individual words.

Although few studies have compared the learning of idioms or other formulaic phrases with familiar and unfamiliar vocabulary, collocations may offer some insight. Kasahara (2011) found that L2 English learners' recall for collocation meanings containing new words was better than recall for the individual words alone (i.e., the L1 Japanese equivalent of *delicious morsel* was better recalled than the Japanese word for *morsel* alone). By combining familiar and unfamiliar words in the target phrases, familiar words (e.g., delicious) serve as cues to better recall the meaning of the phrases (e.g., delicious morsel). Though recent research suggests critical overlap in idioms and collocations (e.g., Bruening, 2020), individual words in collocations differ from idioms critically in their contribution to overall meaning: individual words in collocations often provide additional meaning information, unlike in idioms. Because of this difference, it may neither be the case that individual words are recalled better in idioms nor that the idioms containing unfamiliar words themselves are better remembered. Furthermore, it is also possible that word- and phrase-learning compete with one another when both the phrase and individual words are unfamiliar, and attention to individual words in comparable literal and figurative units may therefore differ between L1 and L2 speakers.

By carefully examining the contribution of figurativeness and individual words in recognition memory for idioms in learners and looking at individual word meaning retention, this study may help shed some light on how idioms are stored during early acquisition processes. In particular, answering the questions outlined by this study may help identify whether the figurativeness of idioms may cause idioms to be stored holistically after only a few exposures and how individual words may interfere or aid in this process.

## Experiment 1: Non-native Speakers

The present study looked at recognition memory for novel phrases that were either learned with a figurative meaning or a literal meaning by non-native speakers. Based on established memory and processing advantages for native speakers, we asked whether the advantage for idioms over literal phrases holds for L2 speakers. Additionally, we explored the interaction between the individual word- and phrasal-meaning during learning by examining the effects of unfamiliar vocabulary on memory for both the phrase and the unfamiliar word. By conducting a training and testing paradigm in which the identical fabricated phrases were either used with an idiomatic meaning or a literal meaning, we could more accurately test differences that are based solely on figurativeness and the impact of the familiarity of constituent vocabulary. Our paradigm used a learning task followed by recognition tasks on form and meaning in addition to a translation task for words. Phrases differed either in meaning (figurative or literal) or by the familiarity of a content noun to language learners (familiar or unfamiliar).

This experiment addresses the first two research questions: 1. Is there a recognition memory advantage for newly learned idioms compared to literal phrases for L2 learners? 2. Does the familiarity of words interact with phrasal learning and affect recognition memory? Considering the first question, we predicted that if L2 learners acquire idioms rapidly as units, like L1 users seem to do (e.g., Reuterskiöld and Van Lancker Sidtis, 2012), recognition of figurative phrases should be better than literal phrases. On the other hand, if figurative meaning poses a challenge unique to L2 learners (e.g., Siyanova-Chanturia et al., 2011), and this disadvantage extends to memory and learning, literal phrases should be better than figurative phrases. Considering the second question concerning the interaction of phrasal- and word-learning in memory for the phrases and/or unfamiliar words, we predicted that it does. However, it may do so in several ways. If the combination of familiar and unfamiliar words improves memory for idiomatic phrases as it does in collocations (e.g., Kasahara, 2011), then the presence of unfamiliar words should improve the recall of phrases overall as both literal and figurative phrases will have the same combination of familiar and unfamiliar words. However, if this effect in collocations is a result of compositional meaning benefits, then this improvement may only be seen in literal phrases. Additionally, if the saliency of this literal word-meaning competes with phrasal meaning (e.g., Cieślicka, 2006), it may decrease recall for only figurative phrases. Finally, considering the recall of the unfamiliar words, we predicted that if less importance is given to these constituents in figurative phrases during learning, as is the case for L1 speakers (e.g., Horowitz and Manelis, 1973), there should be better recall for unfamiliar words in literal phrases compared to figurative phrases.

### Methods

#### Participants

Sixty-five non-native speakers of English (German L1) participated in the study on 2 separate days. Participants (45 female, average age of 25.35, *SD* = 3.11) were given financial compensation for their time and were recruited from the University of Tübingen via university-wide emails. All participants identified as native speakers of German with highly proficient English skills, though their English abilities were somewhat varied. Scores on the LexTale lexical decision task in English (Lemhöfer and Broersma, 2012) varied (range: 45–100, mean 78.75, *SD* = 12.70), and participants' self-reported English ratings averaged across reading, writing, listening, and speaking (on a scale from 1–7, 1 as very poor and 7 as native-like) were 5.49 (*SD* = 0.69, range = 3.75–7). On average, participants reported 7.69 years (*SD* = 4.50) of formal English instruction. Five participants were left-handed.

#### Materials

A total of 30 novel target phrases were developed by the authors for the study, with four variations per phrase. Each phrase had a literal paraphrase and a figurative meaning and also had a variation in which one frequent (familiar) word was replaced with an unfamiliar, less frequent word (e.g., *bell* and *bugle*). The figurative meaning was invented by native speakers as a possible idiomatic meaning for the phrase, though not transparent enough that the meaning is apparent without definition, and the literal meaning was a paraphrase using simple vocabulary (e.g., *the morning bell sounded*, literal: the church bell rang early in the day, figurative: a return from the distraction of day-dreaming; see Familiar/Unfamiliar Noun Selection and Pre-Study: Idiom Material Testing. for norming information). An example phrase with all four variations is displayed in Table 1. The phrases were generally short (mean phrase length in letters: 21.73, in words: 5.06) with no significant differences in length between any four variations. Phrase variations were divided into four counter-balanced lists for learning and testing between participants.

**Table 1:** Example Phrase Stimuli.

**Target Phrase**	**Meaning**	**Noun-type**	**Figurativeness**
The morning *bell* sounded	A return from the distraction of daydreaming	Familiar	Figurative
The morning *bell* sounded	The church bell rang early in the day	Familiar	Literal
The morning *bugle* sounded	A return from the distraction of daydreaming	Unfamiliar	Figurative
The morning *bugle* sounded	The musical horn played early in the day	Unfamiliar	Literal

##### Familiar/Unfamiliar Noun Selection

The familiar and unfamiliar nouns were semantically similar but differed systematically in frequency and their familiarity to the German L2 speaker group tested in this study (familiar or unfamiliar words). Unfamiliar target nouns were first chosen based on their conceptual and semantic comparability to the familiar words (e.g., *bell* and *bugle* are both types of instruments). Additionally, since learners would later be tested on German translations of the nouns, we pre-tested whether native German students in several advanced courses of English studies at the University of Tübingen were able to translate the nouns. Nouns were chosen which did not overlap in form in a substantial way across languages (i.e., English *bell* and the German translation *Glocke* are not cognates) as they differed in their segmental makeup considerably, particularly for the unfamiliar nouns.

A total of 143 nouns were tested on average by 24.86 students of English in four rounds. In the pre-test, students were given a list of up to 60 English nouns and were asked to translate as many of them as possible into German. Included in the list were a mixture of familiar and unfamiliar nouns. Only nouns were selected for Experiment 1 that either all students (familiar) or none (unfamiliar) translated correctly. The subjective familiarity differences identified between the two noun-types were also confirmed with differences in lexical frequencies. *T-*tests showed that known nouns differed systematically in frequency per million (familiar mean 32.20, unfamiliar mean 0.67, *t* = 3.912, *p* < 0.001) based on the SUBTLEX_US_ corpus (Brysbaert and New, 2009). This frequency difference in combination with the pre-test on the target group ensured that the words presented as unfamiliar were indeed unfamiliar to the L2 participants.

##### Pre-study: Idiom Material Testing

In addition to pre-tests for the target nouns in the study, the idioms were also pre-tested as novel idioms to ensure that their meanings were new and not predictable based on their constituent words as well as for their levels of transparency and imageability, which have been shown to affect idiom-learning in native speaker adults (e.g., Steinel et al., 2007; Tiv et al., 2016). Native speakers of English completed three short online tasks (“SurveyGizmo^1^”) to determine (1) whether the meaning of the idiom could be derived only from the parts, (2) a scalar rating for transparency, and (3) a scalar rating for imageability. The tasks were completed in two parts, first meaning and imageability were combined, and second, participants rated the idioms' transparency. The method and instructions closely followed the procedures in Steinel et al. (2007).

Twenty native speakers of English (15 female, average age = 31, SD = 8.54) participated in the 20–30-min online study and were compensated by entry into a gift card lottery. Thirty target idioms with familiar words and 20 filler idioms that were highly familiar for native speakers were included in the online study. Only targets with familiar words were included since the familiar and unfamiliar words are conceptually and visually similar. The filler idioms were included to provide a spectrum of imageable to non-imageable as well as highly transparent to non-transparent idioms and to prevent frustration from interacting with only unfamiliar phrases.

First, participants were asked to give the meaning of the idiom and to rate its imageability. Participants were instructed to paraphrase the idiomatic meaning to the best of their abilities, if known, or to come up with a guess, if possible. Imageability was rated on a scale from 1 to 7 (1: TRUE, 7: FALSE) by rating the truth of the statement: “I could easily visualize this idiom.” Then, in the second part of the survey, participants saw the idiom with a paraphrase of the idiomatic meaning. Transparency was defined as “how related the literal and figurative meanings of the idioms are” and participants rated the truth of the statement: “The figurative meaning of this idiom has a lot in common with its literal meaning.” on a scale from 1 to 7 (1: TRUE, 7: FALSE).

None of the idioms' meanings were guessed by the participants. The idioms were generally rather imageable (range: 2.90–5.95, mean 4.94, *SD* = 0.74). which reflects the choice to include concrete nouns in each idiom. On the other hand, idioms were not very transparent (range: 1.25–4.75; mean: 2.54, *SD* = 1.02), which reflects of the choice to include only idioms with meanings not predictably derived from the literal words. While neither property includes a full range of values, the averages for each idiom are included in additional LMER analyses on the experimental results for each task. Ratings for each idiom are included in the Supplementary Materials.

#### Procedure

##### Learning

The study was conducted in a sound-attenuated room in the LingTüLab at the University of Tübingen on 2 separate days. A learning task was completed on the first day and a short series of testing tasks on the second. On the first day, the LexTale vocabulary test (Lemhöfer and Broersma, 2012) was conducted using “Presentation®^2^,” Software, and the learning task followed using a PowerPoint presentation on a desktop computer. This session took about 20 min in total. The instructions for the learning task were presented in self-guided slides, and the learning task itself was timed using automatically progressing slides. In the instructions, participants were informed that they would be learning the meaning of both literal and figurative phrases, and an example of each was given and presented with the automatic timing to be used in the experiment. Participants were aware that all nouns would also have German glosses but were not instructed to learn any of these words. Participants were also informed that in the next session, they would complete three short tasks testing what they had learned, but no further information on testing procedure was given.

**Figure 1:**
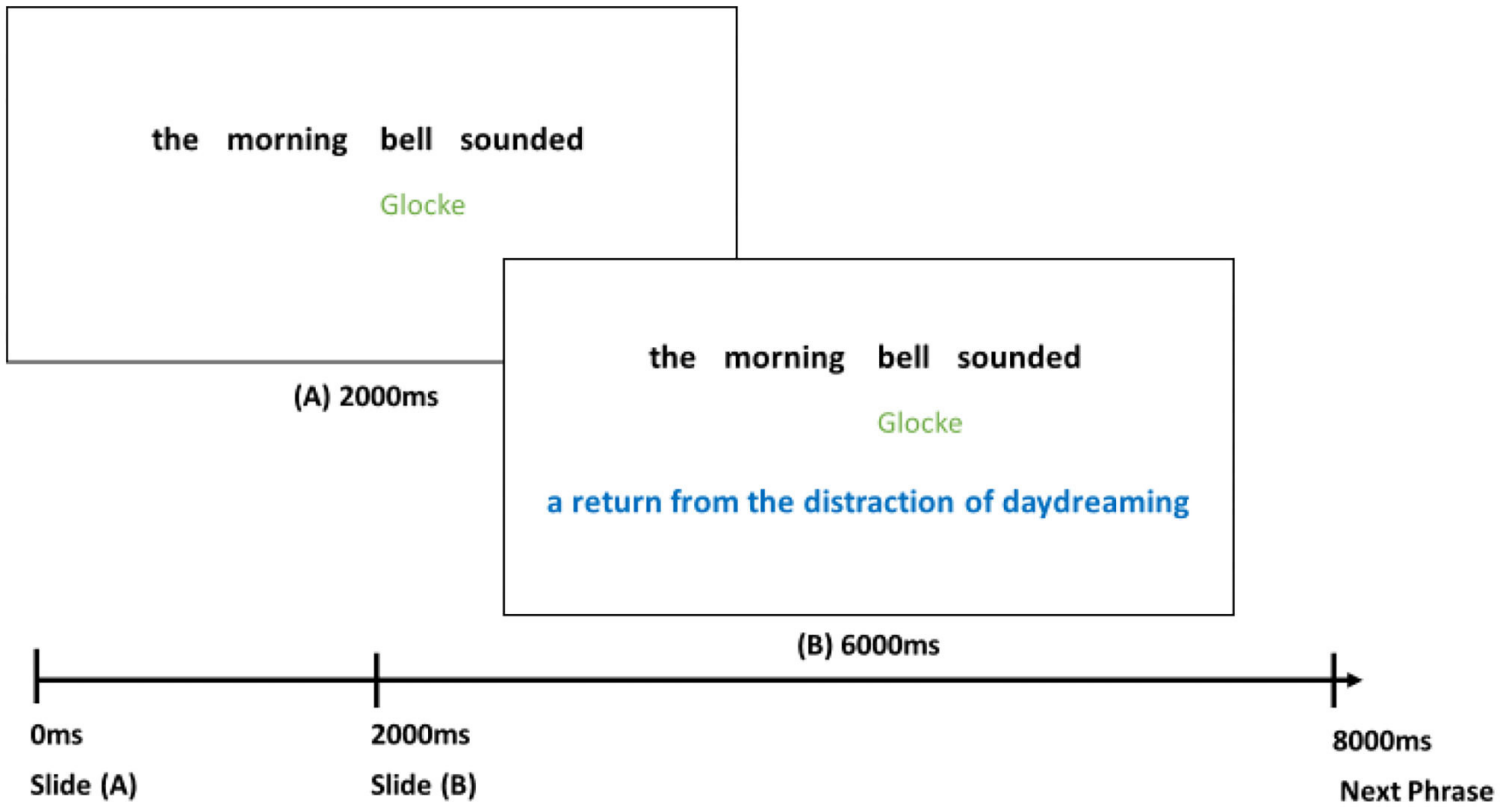
Slide Presentation Timing and Display with Example. The timing of slide presentation displayed from left to right include the phrase in black, German glosses in green, and after 2,000 ms, the meaning of the phrase in blue. In total, the phrase is displayed for the full 8,000 ms before the next phrase appears.

Each participant learned one of the four lists of phrases. The target phrases were presented on slides seen by each participant twice in separate rounds. Each slide contained the target phrase in black, German glosses of all nouns in the phrases in green (maximum of two), and a paraphrase (either literal or figurative) in blue. An example of this presentation is displayed in Figure 1. Each slide was presented for a total of 8,000 ms. Timing ensured participants spent an equal amount of time on each phrase during the learning task. During the first 2,000 ms, only the phrase with German glosses was displayed, and the addition of the paraphrase followed and was presented for an additional 6,000 ms. Once a participant had seen each phrase, a 2-min timed break was imposed, after which participants repeated the procedure again following a single mouse click. The order of phrase-presentation was reversed for half of all participants for each list.

##### Testing

The testing session took place 3 days later, as a rule. However, due to unforeseen scheduling conflicts or illness, four participants were tested 2 days after learning and two participants were tested 4 days later. This interval was chosen after pilot testing with learning and testing on 2 consecutive days showed ceiling performance and null effects. By testing 3 days after the learning session, performance remained high but was no longer at ceiling, providing more optimal conditions for hypothesis testing. Participants returned to the same room in which the learning task was completed. In this testing session, three tasks were conducted consecutively using jsPsych to measure memory differences in: form recognition, meaning recognition, and a translation task. After the completion of the testing tasks, participants completed a language background questionnaire and were then informed more precisely about the phrases they learned.

*Form recognition*. The form recognition task was used to test recognition of the phrases learned during the learning phrase. The test included all 30 phrases (15 idiomatic and 15 literal) presented to each person based on the list they learned in addition to 30 new fillers. All 60 phrases were presented in a randomized order. Fifteen easy fillers were phrases that were lexically different from the learned phrases (7 of them also contained unfamiliar words), and 15 difficult fillers were similar to the learned phrases; difficult fillers were in fact target phrases with the alternative word used from another list (i.e., *the morning bugle sounded* instead of *the morning bell sounded*). In this task, participants were instructed to decide quickly but accurately whether the phrase presented is one of the exact phrases learned in the first session. Responses were measured with a keyboard press marked with green for the dominant hand and red for the non-dominant hand. Participant responses based on correctness were coded for analysis.

*Meaning recognition*. The meaning recognition task tested recognition of the learned meaning of the idiomatic phrases. Participants were presented individually with all 15 idiomatic phrases learned in session 1 and given three multiple choice options. The multiple-choice options included (1) the correct answer, (2) the meaning of another learned idiom, and (3) an idiomatic meaning from an idiom not included in the experiment. For options (2) and (3) only plausible meanings were presented with each idiom. Additionally, a sliding scale was present at the bottom of the screen asking participants how sure they were of their choice (from not at all to very sure). The option “I don't know.” was not included as the bottom of the scale would also indicate this factor. The scale was not numbered, but yielded values from 1 to 100 in the output. Measures of correctness and subjective ratings of sureness were collected for analysis.

*Translation*. The translation task tested learning of the designated target word (familiar or unfamiliar) in the learned phrases. Participants were again presented with all 30 learned phrases individually and asked only to translate the bolded and underlined word back into German (only the target familiar/unfamiliar noun). Specifically, they were asked to try to recall the word given in the first session and instructed to take a guess if unsure or to skip the word if nothing came to mind. Open-ended translations for each target word were recorded and later scored by judges (see Analysis section for details).

### Results

R (R Core Team, 2013) was used to analyze the results of each task individually using mixed effects regression models on the tasks for *Form, Meaning*, and *Translation*. While the analyses differed slightly between tasks, the same procedure was used. *Correctness* (1 = correct, 0 = incorrect) was used as the dependent variable in each case, and, depending on the task, *Phrase-Type* (Figurative or Literal, coded as 0.5 and −0.5, respectively) and *Word-Type* (familiar or unfamiliar, coded as 0.5 and −0.5, respectively) were included as fixed effects. Items and participants were included as random factors with random slopes, where justified. The random effects structure was tested by consulting RePsychLing (Baayen et al., 2015), ensuring that models were sufficiently recognized. In order to account for further variation, factors such as *Trial, LexTale Score*, and *Testing Interval*, numerically centered around zero, were also included in the full models, and backward stepwise selection was used to eliminate these non-theoretically relevant factors in the case that model fit was not improved. Additional analyses were conducted to determine whether the factors *Imageability* and *Transparency* impacted the results. The results of each task will be discussed below individually.

#### Form Results

**Figure 2:**
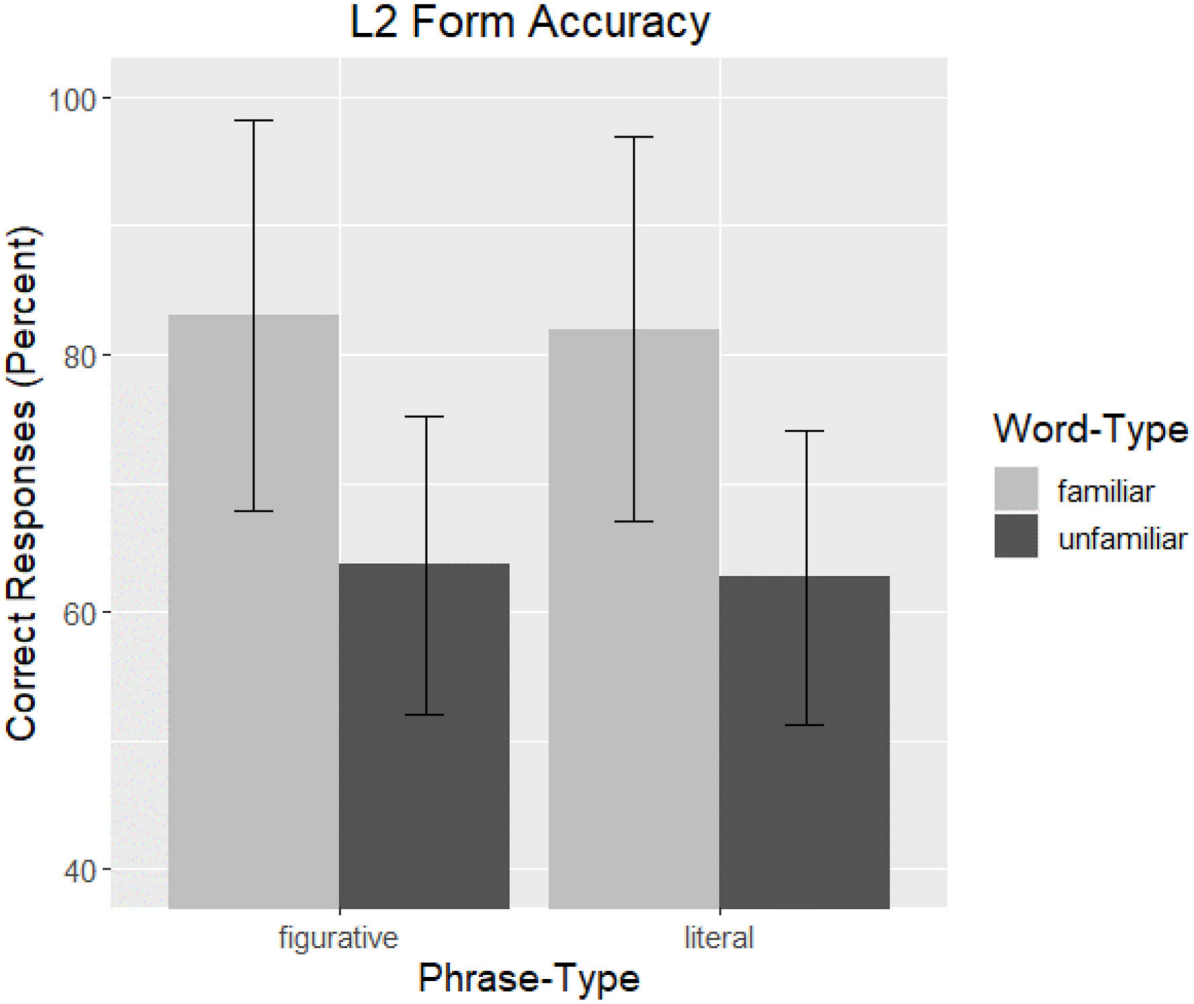
L2 Form Accuracy. The overall correctness of participant responses to form recognition are graphed as a function of *Phrase-Type* by *Word-Type*. Familiar words are represented by light gray and unfamiliar words by dark gray, and bars are the standard error of the mean.

Overall, participants performed well on the task, and total correct responses averaged 82.59% (*SD* = 9.11) across the entire task. Examining target items only, performance was 72.82% (*SD* = 15.68), and participants displayed a wide range of performance averages (30–100%). *Correctness* as a function of *Word-Type* and *Phrase-Type* is graphed in Figure 2.

The estimates from the final LMER model can be seen in Table 2. Of the main factors under investigation, only *Word-Type* was significant (ß = 0.19, *t* = 10.49, *p* < 0.001), and no interaction was present. The effect of *Word-Type* confirms the pattern in Figure 2, namely, that the presence of unfamiliar words significantly decreased accuracy, whereas the type of phrase (figurative or literal) had no effect. The effect of *Word-Type* suggests that a type of competition may be occurring, namely, phrasal- learning is negatively impacted by the presence of unfamiliar words (e.g., Cieślicka, 2006). The lack of a *Phrase-Type* effect is novel, as this effect appears to be robust in L1 speaker groups (e.g., Reuterskiöld and Van Lancker Sidtis, 2012). Though, a lack of interaction does not conform to our expectations.

**Table 2:** L2 form accuracy LMER output.

**Fixed effects**	**β**	***(SE)***	***t***	***Pr(>|t|)***	
Intercept	0.7283	(0.022)	33.097	2.00E−16	***
Phrase-Type	0.0038	(0.018)	0.206	0.837	
Word-Type	0.1933	(0.018)	10.496	2.00E−16	***
Trial	−0.0400	(0.009)	−4.285	1.92E−05	***
Phrase-Type × Word-Type	−0.0018	(0.037)	−0.048	0.962	
**Random effects**	**Variance**	***SD***			
Subject	0.0190	0.138			
Item	0.0032	0.057			

*** *p < 0.001*.

An additional *post-hoc* analysis was conducted on the figurative phrases in order to ensure that the transparency and imageability of the idioms included did not play a role in our results (see e.g., Steinel et al., 2007). In order to do so, the same LMER model, excluding *Phrase-Type* as a factor, was used on the figurative phrases. *Imageability* and *Transparency*, centered around zero, were added to the models, and neither was significant (*Transparency*: ß = 0.00, *t* = −0.05, *p* = 0.963, *Imageability*: ß = −0.01, *t* = −0.38, *p* = 0.704). Furthermore, backward stepwise selection confirmed that they also did not improve model fit. Thus, we conclude that idiomatic differences based on these factors did not affect the results significantly.

#### Meaning Results

The results from the meaning recognition task were recorded in two steps: first, the accuracy of the multiple-choice response (*Correctness*) and second, the ratings on how sure participants were of their choice (*Sureness*). In both cases, responses concerned only the figurative phrases, and therefore only *Word-Type* and not *Phrase-Type* was used as an independent variable in the analyses. While both the correctness and confidence of choice are reported below, the focus will remain on correctness.

##### Correctness

**Figure 3:**
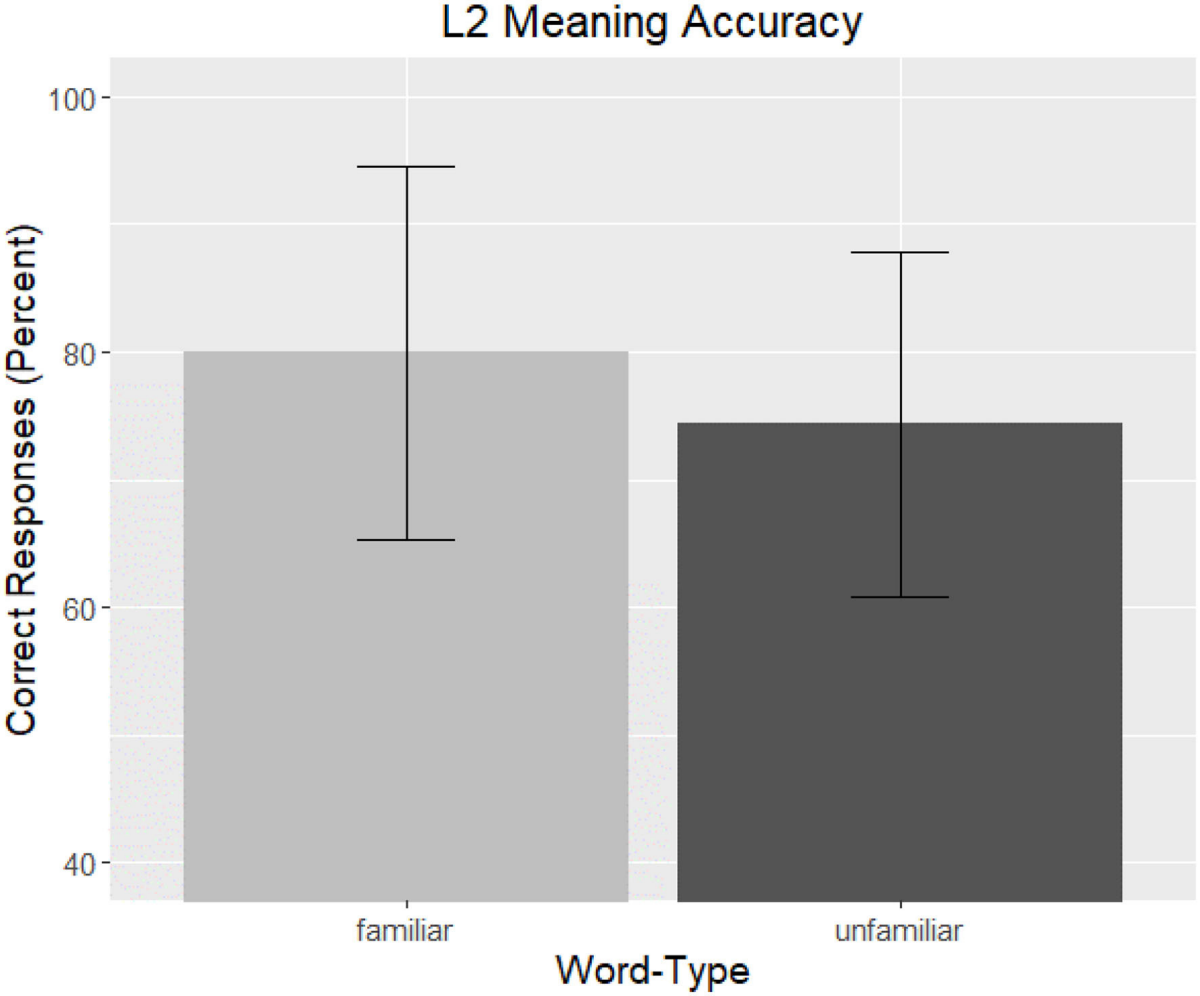
L2 Meaning Accuracy. The overall correctness of participant responses to the meaning of the phrase are graphed as a function of *Word-Type*. Familiar words are represented by light gray and unfamiliar words by dark gray, and bars are the standard error of the mean.

Overall performance on the task was high, and total correct responses averaged 77.13% (*SD* = 17.75) across the entire task. Participants varied widely in their average correctness ranging from 20–100%. Accuracy on the task as a function of *Word-Type* is graphed in Figure 3.

The analysis revealed a significant effect of *Word-Type* (ß = 0.19, *t* = 10.49, *p* < 0.001). No other factors were significant, nor improved model fit, and therefore, the final model included only this factor in addition to items and participants as random factors and slopes. The output of this model can be seen in Table 3. This result replicates those of form recognition in that unfamiliar words decreased performance in meaning recognition.

**Table 3:** L2 meaning accuracy LMER output.

**Fixed Effects**	**β**	***(SE)***	***t***	***Pr(>|t|)***	
Intercept	0.7713	(0.029)	26.902	2.00E−16	***
Word-Type	0.0563	(0.024)	2.305	0.0214	*
**Random effects**	**Variance**	***SD***			
Subject	0.0221	0.149			
Item	0.0100	0.100			

* *p < 0.05*,

*** *p < 0.001*.

Additionally, the factors *Imageability* and *Transparency* were neither significant, nor did they improve model fit (*Transparency*: ß = 0.02, *t* = 1.08, *p* = 0.286, *Imageability*: ß = 0.01, *t* = 0.71, *p* = 0.481). Thus, these factors did not play a significant role in the participants performance on meaning recognition, as in form recognition.

##### Confidence of Choice

Confidence of choice (*Sureness*) was measured on a sliding scale (1–100) and was selected with the multiple-choice answer used in the above analysis. Participants were generally confident in their choices, and the average sureness rating was 69.99% (*SD* = 30.92), though there was wide variability in this choice (range: 0–100).

While this measure was not the focus of our investigation, we also conducted LMER models on this score to determine if confidence of choice simply aligns with correctness, or whether the word-type also impacted this choice independently. In our model, we used *Word-Type* and *Correctness* (coded as −0.5 for incorrect, 0.5 for correct) as our independent predictors. The output of this model can be seen in Table 4. The analysis confirms the results reported on accuracy: both *Word-Type* (ß = 9.07, *t* = 4.85, *p* < 0.001) and *Correctness* (ß = 24.17, *t* = 11.72, *p* < 0.001) were significant predictors, though the effect size for *Correctness* was greater than *Word-Type*. No interaction was present. These results suggest that participants were overall more confident in their choices when they were correct but also when there were only familiar words present; unfamiliar words decreased overall sureness independent of the correctness of their choice.

**Table 4:** L2 meaning sureness LMER output.

**Fixed effects**	**β**	***(SE)***	***t***	***Pr(>|t|)***	
Intercept	63.389	(2.248)	28.201	2.00E−16	***
Word-Type	9.073	(1.868)	4.858	1.4E−06	***
Correctness	24.170	(2.061)	11.727	2.00E−16	***
Correctness × Word-Type	3.401	(3.825)	0.889	0.374	
**Random effects**	**Variance**	***SD***			
Subject	146.930	12.121			
Item	56.340	7.506			

*** *p < 0.001*.

#### Translation Results

In the translation task, participants were asked to provide a translation from English into German for one target word in each phrase. In doing so, they were asked to try to reproduce the gloss shown in the learning phrase, if possible. The responses were first scored as correct or incorrect by hand, and *Correctness* was subsequently analyzed for the translations of unfamiliar words based on *Phrase-Type*. The processes of scoring analysis are described below.

##### Scoring

All responses were scored, including translations of both familiar and unfamiliar words. Answers were categorized as correct (1) or incorrect (0). The judges were two native speakers of German with highly proficient English skills. Where the two scorers disagreed, a third L1 judge made the decision. Answers were scored as correct if they were one of the following: a match with the definition given in the learning task; a conceptually correct, close synonym with the word given in the learning task (e.g., *Armreif* and *Armband*, English: bangle); a partial match that was underspecified (e.g., *Fischschwarm* and *Schwarm*, English: shoal); or misspelled or abbreviated versions of the correct word (e.g., *Pullover* and *Pulli*, English: sweater). All other responses were marked as incorrect.

##### Correctness

Overall, participants did well on the task despite not being given any direct instructions on or indication of a word-learning task, and they had an average of 78% (*SD* = 17.88) correct translations. Additionally, performance on the familiar (mean: 97.02%, *SD* = 5.89) and unfamiliar words (mean: 33.33%, *SD* = 22.32) was significantly different (*t* = 39.67, *p* < 0.001), a result in line with pre-tests suggesting that the familiar words were indeed highly familiar compared to the unfamiliar words.

**Figure 4:**
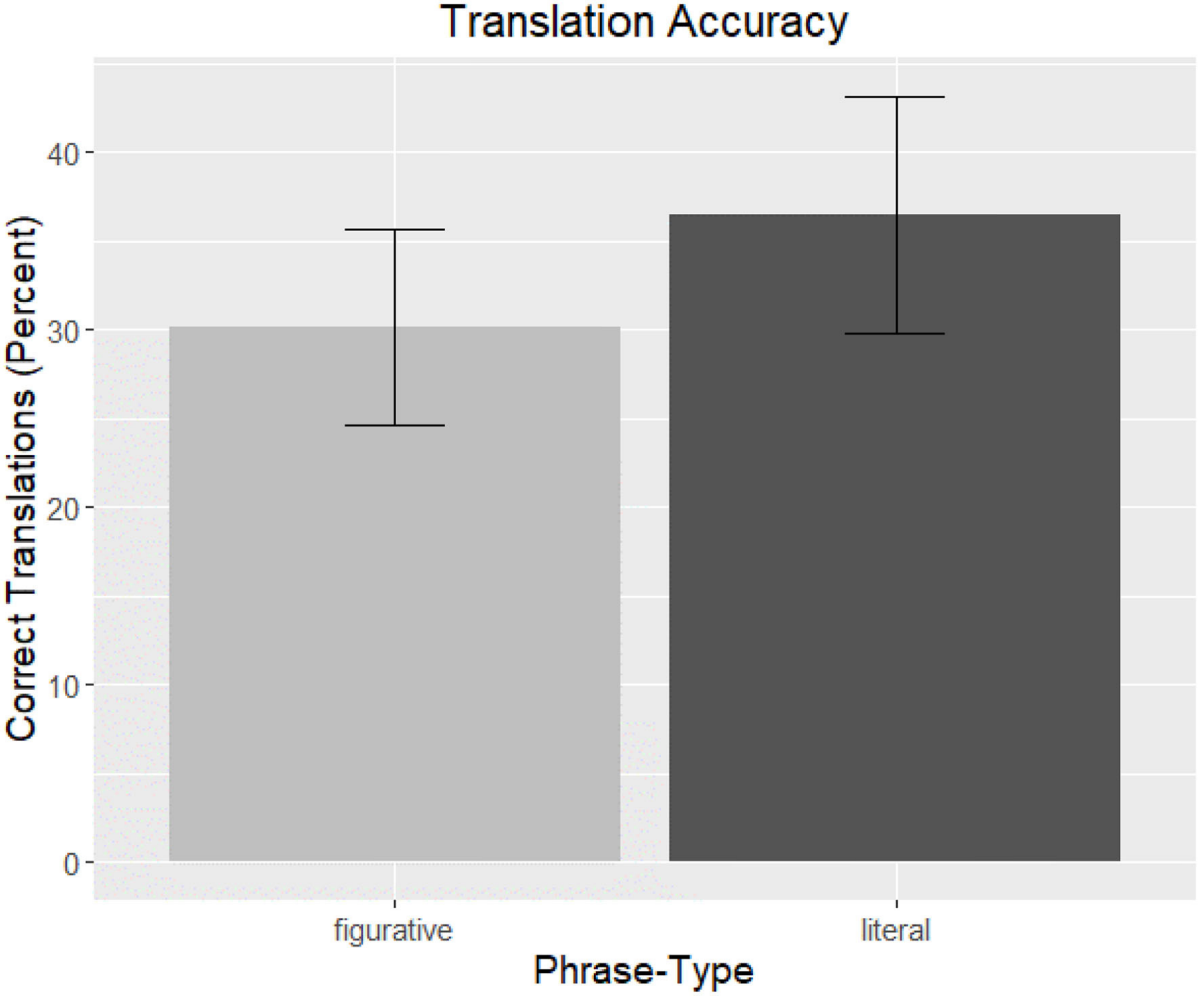
L2 Translation Accuracy. The overall correctness of participant translations of unfamiliar words are graphed as a function of *Phrase-Type*. Translations occurring in figurative phrases are represented by light gray and literal phrases by dark gray, and bars are the standard error of the mean.

The analysis again used *Correctness* as the dependent variable, and *Phrase-Type* as an independent factor. In addition to the factors of *Trial, LexTale Score*, and *Testing Interval, Word Frequency* (based on word form frequency from Brysbaert and New, 2009, also centered around zero) was also used in the full model to account for any additional variation based on frequency within this group of unfamiliar words. The output of the final model can be seen in Table 5. Only *Phrase-Type* (ß = −0.05, *t* = −2.20, *p* < 0.05) and *LexTale Score* (ß = 0.08, *t* = 3.31, *p* < 0.01) remained in the final model, and both factors were significant. As shown in the graph in Figure 4, unfamiliar words were more accurately translated when they were encountered as part of a literal phrase compared to figurative phrases. Additionally, higher LexTale scores predicted better performance on the translation task.

**Table 5:** L2 translation accuracy LMER output.

**Fixed effects**	**β**	***(SE)***	***t***	***Pr(>|t|)***	
Intercept	0.333	(0.041)	8.212	8.36E−11	***
Phrase-Type	−0.056	(0.025)	−2.207	0.0276	*
LexTale score	0.085	(0.026)	3.316	0.0015	**
**Random effects**	**Variance**	***SD***			
Subject	0.032	0.179			
Item	0.030	0.173			

* *p < 0.05*,

** *p < 0.01*,

*** *p < 0.001*.

Additionally, the factors *Imageability* and *Transparency* were applied only to the idiomatic targets, to see whether these factors may have impacted the results of the figurative phrases. They neither improved model fit nor were significant (*Transparency*: ß = 0.02, *t* = 0.66, *p* = 0.514, *Imageability*: ß = 0.01, *t* = 0.35, *p* = 0.724). Thus, we confirm that these factors did not play a role in participants' performance on the translation task.

### Interim Discussion

In the first experiment, we compared figurative and literal readings of identical phrases learned with equal exposure. We did not find evidence of memory differences based on the figurativeness of a phrase. These results may either suggest that L2 learners, in contrast to L1 speakers, do not acquire figurative language rapidly in a manner different than literal language, or they suggest that there is generally no memory advantage when identical phrases are compared. In the case of the latter, L1 speakers should also show no memory advantage for the materials of Experiment 1. While Reuterskiöld and Van Lancker Sidtis (2012) did find evidence of this advantage in L1 children, their study used low-frequency idioms and surveyed the parents of the children involved on their knowledge and use of the idioms in question, and it may be the case that the children were exposed to the idioms at some point before the experiment. Additionally, as exposure happened in a naturalistic setting, phrases may have also been presented differently (i.e., with differing prosodic cues) in a manner that could not be controlled for in their study. Thus, additional evidence is needed in order to determine whether this effect applies to both learner groups.

Considering the competition between phrasal and word meaning, we found that unfamiliar words impacted phrasal learning. Critically, this impact did not differ between figurative and literal phrases. We interpret this decrease in performance in phrases with unfamiliar words to a more divided attention on word- and phrasal-meaning, even where literal word meaning is irrelevant. Although participants were asked only to “learn the meaning of the phrase,” the presence of an unfamiliar word still served as a salient cue, and more attention may have been given to the German glosses while these phrases were being presented. Critically, however, the glosses were present for all nouns in all phrases, so the difference should have been caused by the word's familiarity rather than the glosses. We also found that individual words were learned better when they were part of a literal phrase as evidenced by the translation task results. This effect may be in line with the L1 recognition results found by Horowitz and Manelis (1973) in which similar effects were found with unitized compared to non-unitized word pairs. Thus, while figurativeness did not impact the recall of the phrases, it did impact how well translations of unfamiliar words were recalled.

Overall, the negative result concerning memory differences between learned phrases based on figurativeness alone leaves questions unanswered. In particular, whether this lack of effect is unique to L2 learners or whether it also holds for adult L1 speakers where identical phrases and learning environments are used. In order to determine whether this is the case, we conducted a follow-up experiment using the same phrases and procedure on adult native speakers.

## Experiment 2: Native Speakers

Experiment 2 replicates Experiment 1 for native speakers, and it asks whether idiomatic advantages for adult native speakers extend to recognition memory when phrases are controlled for vocabulary and exposure. If L1 speakers do show advantages for idioms compared to literal phrases, then we expect better performance for figurative compared to literal phrases in the recognition tasks. Such a result would indicate a difference in storage and processing between L1 and L2 speakers. If, however, again no memory advantages are found, then this may suggest a more general effect of the experimental item control and learning conditions of the experiment.

Although not the focus of Experiment 2, we will also examine how the presence of unfamiliar words affects phrasal learning in native speakers. For native speakers, unfamiliar words are low-frequency, but not necessarily unknown or entirely unfamiliar as they are for L2 speaker participants. However, for consistency, this term will also be used for the L1 experiment, and this difference is considered in the interpretation of the results. While it may be the case that the presence of less frequent words negatively impacts L1 phrasal learning similarly to L2 phrasal learning, it may also be the case that unfamiliar words are more unexpected for L1 speakers, and, in contrast to L2 speakers, these words serve as highly salient cues. In this case, we would expect an improvement of recall for either all phrases with such words, or an interaction following our last prediction.

### Methods

#### Participants

Twenty-five native speakers of English participated in the study on 2 separate days. Participants (14 female, average age of 31.76, *SD* = 11.04) were given financial compensation for their time and were recruited from the University of Tübingen via university-wide emails. At the time of the study, participants had lived in Germany for an average of 5.83 years (*SD* = 5.87) but still reported using English an average of 74% of the time in their day-to-day lives (SD = 21.27). Two participants were left-handed.

#### Materials

The materials used were the same as in Experiment 1.

#### Procedure

##### Learning

The study was conducted, as in Experiment 1, in a sound-attenuated room in the LingTüLab at the University of Tübingen on 2 separate days including a learning task on the first day and a testing task on the second. Unlike in Experiment 1, participants were told that the figurative phrases were examples of idioms taken from other languages or varieties of English. This would ensure that these participants also believed themselves to be learning existing idiomatic phrases. Participants were also informed that in the next session, they would complete two short tasks testing what they learned, but no further information was given.

As in Experiment 1, participants learned one of the four lists of phrases. The target phrases were presented on slides seen by each participant twice. Each slide contained the target phrase in black and a paraphrase (either literal or figurative) in blue. The only visual difference from Experiment 1 was the exclusion of German glosses for nouns. All timing remained the same.

##### Testing

The testing session took place 3 days later for all participants. Participants returned to the same room in which the vocabulary was learned for testing. In this session, only two of the three tasks from Experiment 1 were conducted consecutively using jsPsych to measure learning: form recognition and meaning recognition. The translation task was not included, as no translations were presented during the learning experiment. After the completion of the testing tasks, participants completed a language background questionnaire and were then informed more precisely about the phrases they learned.

Both form and recognition tasks were identical to Experiment 1.

### Results and Discussion

R (R Core Team, 2013) was used, as in Experiment 1, to analyze the results of each task individually using mixed effects regression models on the tasks for *Form* and *Meaning*. Coding for the dependent variable *Correctness* (1 = correct, 0 = incorrect) as well as the factors of *Phrase-Type* (Figurative or Literal, coded as 0.5 and −0.5, respectively), *Word-Type* (familiar or unfamiliar, coded as 0.5 and −0.5, respectively), and *Trial* (numerically centered around zero) were identical to Experiment 1. The results are discussed by task below.

#### Form Results

**Figure 5:**
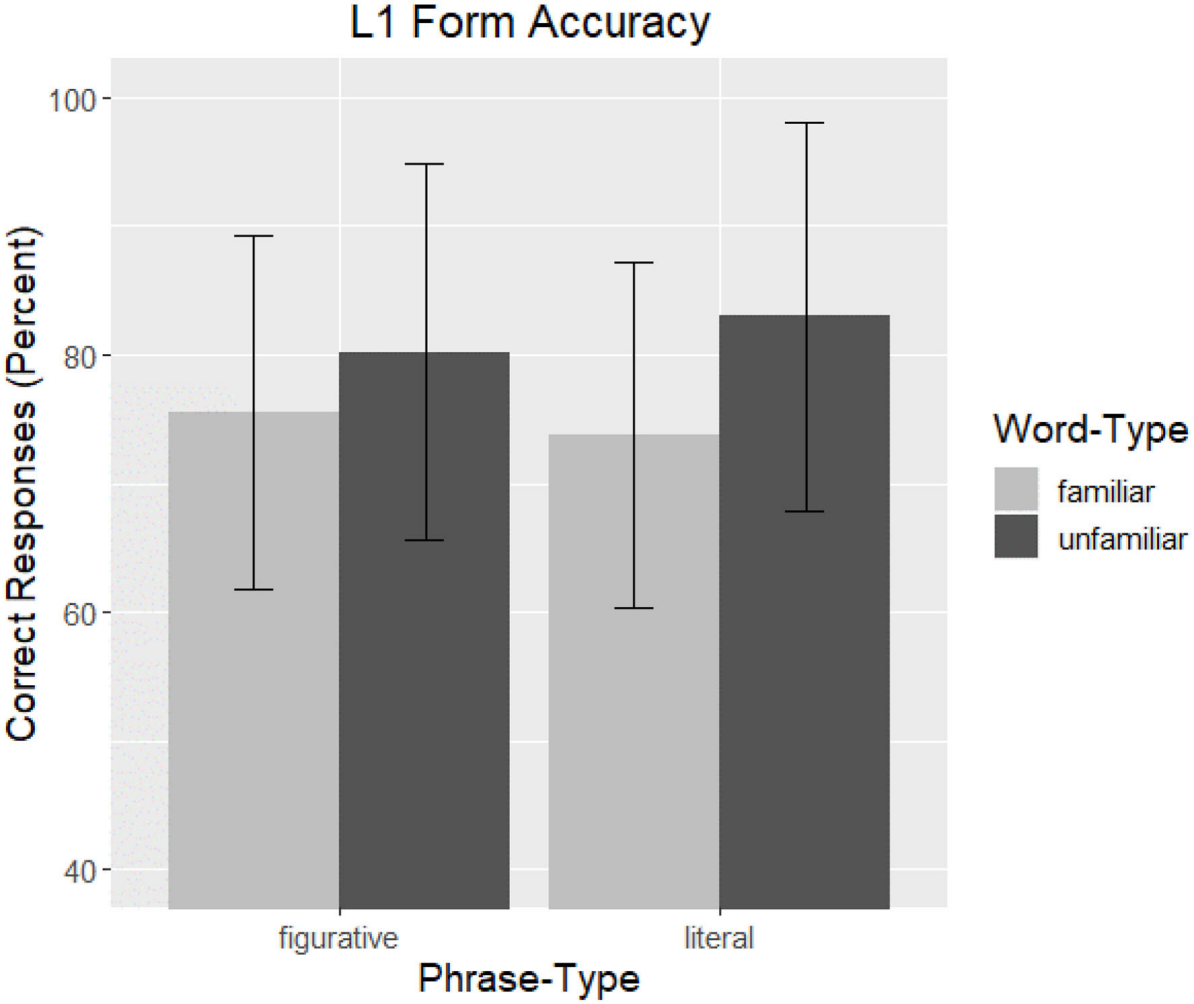
L1 Form Accuracy. The overall correctness of participant responses to form recognition are graphed as a function of *Phrase-Type* by *Word-Type*. Familiar words are represented by light gray and unfamiliar words by dark gray, and bars are the standard error of the mean.

Overall, total correct responses averaged 85.60% (*SD* = 8.19) across the entire task, slightly higher than L2 performance in Experiment 1 (82.59%). On target items, performance was 78.13% correct (*SD* = 15.95), and participants again displayed a wide range of performance averages (33.33–96.66%). Overall correctness as a function of *Word-Type* and *Phrase-Type* is graphed in Figure 5.

The final LMER model estimates can be seen in Table 6. As in Experiment 1, *Word-Type* showed the trend of a main effect, though it was not statistically significant (ß = −0.06, *t* = −1.74, *p* = 0.09), and neither an effect of *Phrase-Type*, nor an interaction was present. The effect of *Word-Type* displays the trend seen in Figure 5, namely, that the presence of an unfamiliar word may increase accuracy, whereas the type of phrase (figurative or literal) had no effect. While the trending effect of *Word-Type* showed the opposite pattern displayed by L2 learners in Experiment 1, the lack of effect of *Phrase-Type* replicates the pattern in Experiment 1.

**Table 6:** L1 form accuracy LMER output.

**Fixed effects**	**β**	***(SE)***	***t***	***Pr(>|t|)***	
Intercept	0.7809	(0.033)	23.572	2.00E−16	***
Phrase-Type	−0.0096	(0.028)	−0.348	0.7280	
Word-Type	−0.0650	(0.037)	−1.749	0.0935	
Trial	−0.0506	(0.014)	−3.585	3.60E−04	***
Phrase-Type × Word-Type	0.0536	(0.055)	0.971	0.33	
**Random effects**	**Variance**	***SD***			
Subject	0.0026	0.051			
Item	0.0205	0.143			
Word-Type	0.1251	0.570			

*p < 0.10*,

*** *p < 0.001*.

As in Experiment 1, we conducted an additional *post-hoc* analysis on the figurative phrases in order to ensure that the transparency and imageability of the idioms included did not influence the results significantly (see e.g., Steinel et al., 2007). Again, the same LMER model, excluding *Phrase-Type* as a factor, was used on the figurative phrases. Imageability and Transparency, centered around zero, were added to the models, and neither were significant, nor did they change the findings in our full models (*Transparency*: ß = 0.00, *t* = 0.17, *p* = 0.864, *Imageability*: ß = 0.01, *t* = 0.92, *p* = 0.361). Furthermore, backward stepwise selection confirmed that they also did not improve model fit. Thus, we conclude that idiomatic differences based on these factors did not affect the results.

#### Meaning Results

As in Experiment 1, both accuracy of the multiple-choice response (*Correctness*) and ratings on how confident participants were in their choice (*Sureness*) were measured and will be discussed below. Only *Word-Type* was used as an independent variable in the analyses as the task concerned only figurative phrases. Like Experiment 1, the focus will remain on accuracy.

##### Correctness

**Figure 6:**
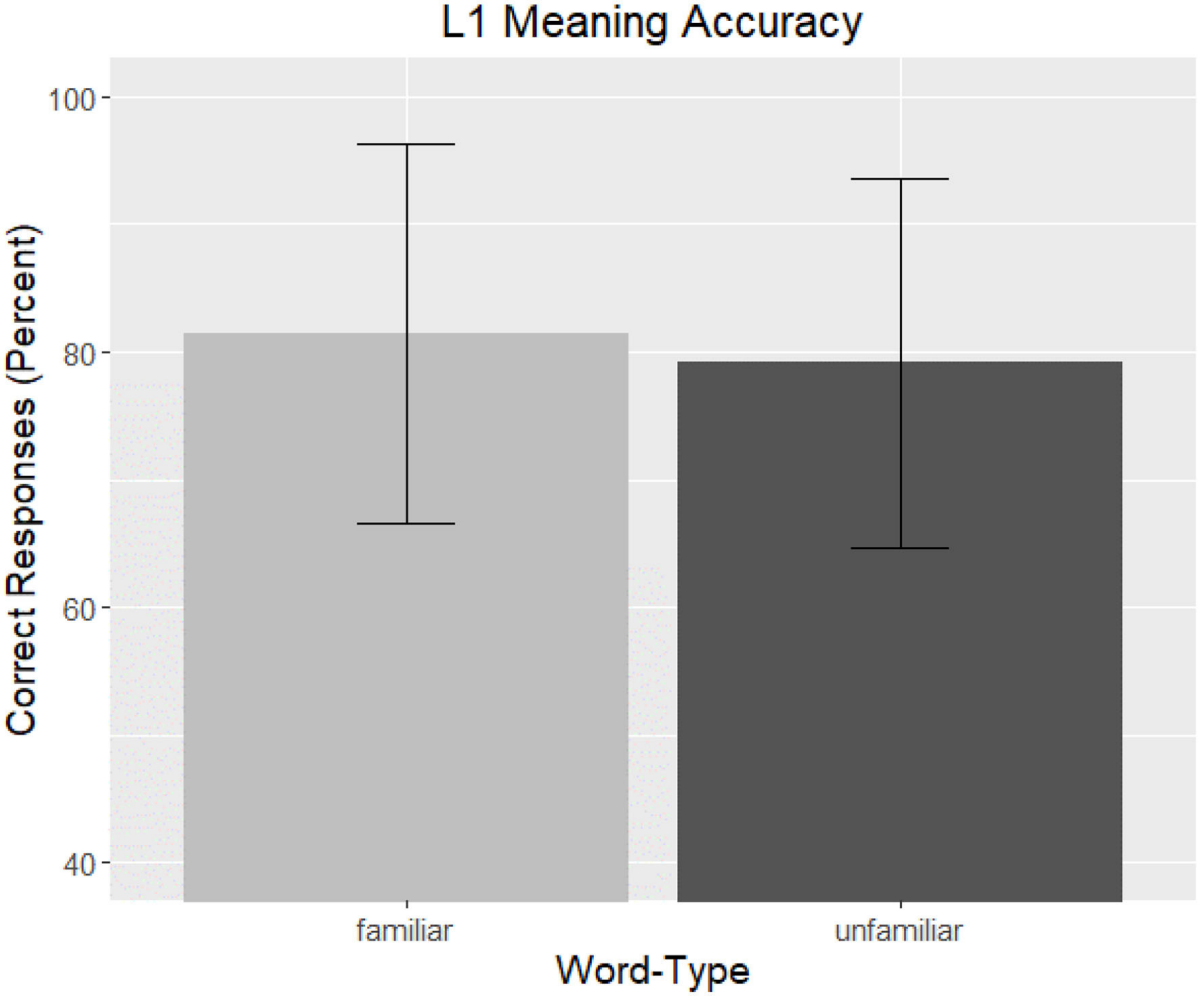
L1 Meaning Accuracy. The overall correctness of native speaker participant responses to the meaning of the phrase are graphed as a function of *Word-Type*. Familiar words are represented by light gray and unfamiliar words by dark gray, and bars are the standard error of the mean.

Correct responses averaged 80.26% (*SD* = 18.40) across the entire task. Participants varied widely in their average correctness ranging from 33.33–100%. Accuracy on the task as a function of *Word-Type* is graphed in Figure 6.

The analysis revealed no significant effects, though a marginal effect of *LexTale Score* was present. Unlike the results of the Form recognition task and the L2 results, *Word-Type* was not a significant factor. The output of the final model can be seen in Table 7.

**Table 7:** L1 meaning accuracy LMER output.

**Fixed effects**	**β**	***(SE)***	***t***	***Pr(>|t|)***	
Intercept	0.8028	(0.042)	8.912	2.00E−16	***
Word-Type	0.0277	(0.037)	0.743	0.4660	
**Random effects**	**Variance**	***SD***			
Subject	0.0256	0.1600			
Item	0.0137	0.1170			
Word-Type	0.0485	−0.6900			

*p < 0.10*,

*** *p < 0.001*.

Additionally, the factors *Imageability* and *Transparency* were added and checked with model comparison, as in Experiment 1. Neither factor was significant, nor improved model fit (*Transparency*: ß = 0.03, *t* = 1.36, *p* = 0.184, *Imageability*: ß = 0.01, *t* = 0.69, *p* = 0.495). Thus, these factors did not play a role in the L1 and L2 participants' performances on meaning recognition, as in form recognition.

##### Confidence of Choice

L1 participants were also generally confident in their choices, and the average sureness rating was 75.91% (SD = 27.42), and participants used the entire range of the scale (range: 0–100).

We conducted LMER models on this score using *Word-Type* and *Correctness* (coded as −0.5 for incorrect, 0.5 for correct) as our independent predictors. The output of this model can be seen in Table 8. The analysis confirmed the results reported on accuracy: only *Correctness* (ß = 25.93, *t* = 8.68, *p* < 0.001) was a significant predictor, not *Word-Type*. No interaction was present. These results suggest that participants were overall more confident of their choices when they were correct. As in the L1 accuracy results, and unlike the L1 trend in the results for meaning recall, *Word-Type* does not seem to affect the accuracy of meaning recall for native speakers.

**Table 8:** L1 meaning sureness LMER output.

**Fixed effects**	**β**	***(SE)***	***t***	***Pr(>|t|)***	
Intercept	68.0517	(3.042)	22.369	<2.00E−16	***
Word-Type	0.6897	(2.618)	0.263	0.7920	
Correctness	25.9341	(2.987)	8.683	<2.00E−16	***
Word-Type × Correctness	2.0271	(5.418)	0.374	0.71	
**Random effects**	**Variance**	***SD***			
Subject	136.16	11.669			
Item	58.48	7.647			

*p < 0.10*,

*** *p < 0.001*.

### General Discussion

Neither Experiment 1 nor Experiment 2 showed evidence of recognition memory differences in phrasal recall based on the figurativeness of a phrase. Thus, the lack of recognition memory difference between the two phrase-types is not unique to L2 learners. Although Reuterskiöld and Van Lancker Sidtis (2012) compared memory for literal and figurative phrases in L1 children following a single exposure (e.g., *cross swords with someone* and *in a new school*) and found better recall of the new idioms, the possibility of previous encounters with the idiomatic phrases cannot be entirely discounted. Thus, a “rapid uptake” (pp. 221) of idiomatic phrases cannot be attributed to the figurativeness of the idiom alone. One possible reason for the difference in results may be based on the method of exposure to the idioms. Whereas, the current study used visual presentation with a focus on phrasal learning, exposure to the phrases in the previous study occurred auditorily in a naturalistic setting in which experimenters used the phrases as part of normal communication during an activity. As the authors point out, prosody may be a crucial component of retaining formulaic language such as idioms, and these cues may have unintentionally played a role in signaling the idiomatic phrases more prominently (see e.g., Van Lancker et al., 1981). Additionally, it may be the case that natural acquisition such as that explored in the Reuterskiöld and Van Lancker Sidtis may differ from the type of learning tested in the current experiment which may be more reminiscent of formal educational settings.

Another possible reason that the current study did not show evidence of recognition differences between literal and figurative phrases of equal nature is that they were both learned as phrases. Previous studies which found differences in processing for L1 speakers comparing idiomatic phrases to novel literal phrases typically compared similar, though not equal phrases (e.g., *at the end of the day* vs. *at the end of the war*) or phrases with slight changes in word order (e.g., *hit the nail on the head* vs. *hit his head on the nail*). These novel phrases may have been encountered as phrases for the first time or as one of very few exposures (e.g., Conklin and Schmitt, 2008; Siyanova-Chanturia et al., 2011). In contrast, and more consistent with the current results, when comparing literal and figurative readings of the same phrases, these studies did not all find differences between reading times, though the phrases were familiar in such studies. This result is also in agreement with research from Tabossi et al. (2009) comparing the processing of idioms and known clichés to matched controls. In a recognition task, participants showed differences between matched controls and both types of formulaic language, but idioms did not differ from clichés. The authors concluded that idioms have processing advantages because of their familiarity and not due to a holistic storage or their figurative nature. As both types of phrases in the current study were equally familiar based on exposure in the learning phase, our results would also be consistent with this characterization of idiomatic processing. However, it should not be discounted that such an interpretation assumes that the advantages present in processing transfer directly to memory.

Experiment 2 also showed a statistically non-significant trend in differences in recall based on the presence of unfamiliar (infrequent) words, though the direction of this effect was in opposition to the significant L2 findings in Experiment 1. Namely, whereas L2 learners showed significantly poorer recall of the phrase where unfamiliar words were present, L1 speakers showed a tendency toward better recall when unfamiliar words (less frequent words) were present. Yet, this effect did not significantly impact the recall of the meaning of the phrase for L1 speakers as it did for L2 learners. This effect and lack of interaction, like Experiment 1 in nature though not direction, suggests that properties of the individual words may affect the processing of the entire phrase, regardless of figurativeness. This overall result is in line with findings from Arnon and Cohen Priva (2015), in which word frequency impacted the phrasal duration of multi-word sequences, even when these phrases were highly frequent. Namely, individual word properties may continue to affect phrasal processing, even after phrases are learned as such. Concerning the direction of this effect, there may be different forces at work for each learner group. For the L2 learners in Experiment 1, the saliency of literal word meanings during learning may have created competition between phrasal- and word-learning, regardless of figurativeness (e.g., Giora, 1997). In the case of unfamiliar words, the ability to ignore their non-salient meaning may be overridden as the saliency of these words is by default greater for this group of learners. This finding is in line with research suggesting that L2 learners may rely on individual words during learning overall and have more difficulty building multi-word units (e.g., Arnon and Christiansen, 2017). For the L1 speakers in Experiment 2, we expect that while the unfamiliar words were infrequent, they were not entirely unknown. Rather than having the challenge of learning both phrasal- and word-meanings (the latter of which was not provided for L1 speakers), the L1 speakers could focus more on the phrase as a whole, and the surprise of unfamiliar L1 words may have caused the phrases containing such words to be more salient than other phrases. Rather than competition between word- and phrase-learning, as we suggest took place in the L2 learners, the presence of such words tended to increase recall as L1 speakers noticed but did not need to learn these words. However, this claim needs further research, as this trend was not statistically significant.

Finally, though there was no evidence of memory advantages for figurative phrases in the recall tasks in Experiments 1 and 2, the results of the translation task in Experiment 1 suggest that there may still be differences in the recall of word meaning based on the figurativeness of the phrase. L2 learners better recalled the German translations of unfamiliar words when they were part of literal phrases. While this effect seems in line with the L1 recognition results found by Horowitz and Manelis (1973) in which individual words in non-unitized (cold egg) compared to unitized word pairs (cold war) were recalled better, we do not suspect that this recognition memory effect is due to a difference in storage or unitization between literal and figurative phrases. Had this been the case, we would have also expected differences in recall on this basis, as originally predicted. Indeed, the effect found by Horowitz and Manelis might also be explained based on differences in exposure to the phrases used, which is not the case in the current study. Rather, the result from our L2 learners may be more in line with the collocation results suggested by Kasahara (2011). The individual words in literal phrases from the current study were learned as part of (literal) phrases not unlike the way collocations are learned. In this manner, the unfamiliar words were part of a phrase containing familiar words in both figurative and literal phrases, but the unfamiliar words in literal phrases may have been better recalled due to this compositional meaning effect (i.e., all words contributed to the phrasal meaning). For the unfamiliar words in figurative phrases, as the words did not contribute to the phrasal meaning, such an effect is not present. Thus, there is some evidence that figurativeness impacts the learning of individual words within phrases.

Overall, in comparing the results between Experiment 1 and Experiment 2 and their implications on native- and non-native speakers' idiom-learning, it is important to point out that the evidence does not point to general differences in the acquisition of figurative phrases between speaker groups. On the contrary, the overall similar performance in recognition recall based on form and meaning recall suggests that L1 and L2 speakers likely learn phrases—figurative and literal—in the same manner. If indeed figurative language poses a burden during processing as some literature suggests (e.g., Siyanova-Chanturia et al., 2011), it does not appear to do so for recognition memory. Rather, this is in line with findings suggesting that processing, and here recognition memory, is similar in L1 and L2 speakers (e.g., Conklin and Schmitt, 2008; Beck and Weber, 2016a). The differences between the two speaker groups suggested by the presence of new or unfamiliar vocabulary reflects general differences based on L1 and L2 language experience. While the recognition memory effects from the phrasal recall tasks did not confirm a saliency-based non-native focus on constituent words rather than phrases in figurative phrases (e.g., Cieślicka, 2006; Durrant and Schmitt, 2009), the retention of word-meanings did tend to differ between the two phrase types, and additional studies may be needed in order to follow-up on these assumptions. Furthermore, without comparable L1 data, it is unclear whether this applies to all language learners as collocational learning may suggest or merely L2 language learners. Critically, though, constituent words did impact recognition recall in both groups, and we predict that language experience dictated the direction of that effect on recall.

## Conclusion

Motivated by previous research suggesting a difference in the acquisition and storage of idiomatic phrases compared to literal phrases (e.g., Reuterskiöld and Van Lancker Sidtis, 2012), the current study set out to examine the presence of this difference first in non-native learners and then in adult native speakers. Additionally, we aimed to examine the role of constituent words on phrasal learning, with a focus on L2 learners. We found that recognition memory for idiomatic phrases does not differ from literal phrases when these phrases are identical, and this holds for both native and non-native speakers. Additionally, phrasal meaning does interact with the presence of unfamiliar constituent words in both types of phrases, but it seems to do so differently for L1 and L2 learners. While the presence of unfamiliar words shows a trend of boosting recognition memory for figurative and literal phrases in native speakers, it significantly decreases overall recognition for non-native speakers. Furthermore, non-native speakers show more learning of these unfamiliar words in literal compared to figurative phrases.

We conclude that idioms are not necessarily stored differently from literal phrases after a single learning exposure session. If holistic storage of idioms is systematically different from literal language and this difference is based on figurativeness alone, a single learning session with visual stimuli is insufficient in producing measurable differences. Future studies should continue to investigate memory differences between phrases more equal in vocabulary and exposure using a variety of training and testing measures in order to better evaluate whether figurativeness or unitization may account for differences in findings.

## Data Availability Statement

The raw data have been archived in TALAR (Tübingen Archive of Language Resources) at: https://hdl.handle.net/11022/0000-0007-EB23-9 and are available by request.

## Ethics Statement

Ethical review and approval was not required for the study on human participants in accordance with the local legislation and institutional requirements. The patients/participants provided their written informed consent to participate in this study.

## Author Contributions

SB was primarily responsible for the conception, design, and analyses of the results with support and input from AW throughout each stage in the process. The interpretation of the results was done in collaboration between both authors. SB was primarily responsible for drafting the text and the final version was revised by both authors. Both SB and AW approved the submitted text and can be held accountable for aspects of accuracy and integrity.

## Conflict of Interest

The authors declare that the research was conducted in the absence of any commercial or financial relationships that could be construed as a potential conflict of interest.

## Footnotes

^1^SurveyGizmo. Boulder, Co., USA. Retrieved from: www.surveygizmo.com.

^2^Presentation^®^. Berkely, CA: Neurobehavioral Systems, Inc. Retrieved from: www.neurobs.com.
